# Examining the Link between Biofilm Formation and the Ability of Pathogenic *Salmonella* Strains to Colonize Multiple Host Species

**DOI:** 10.3389/fvets.2017.00138

**Published:** 2017-08-25

**Authors:** Keith D. MacKenzie, Melissa B. Palmer, Wolfgang L. Köster, Aaron P. White

**Affiliations:** ^1^Vaccine and Infectious Disease Organization-International Vaccine Centre, Saskatoon, SK, Canada; ^2^Department of Microbiology and Immunology, University of Saskatchewan, Saskatoon, SK, Canada; ^3^Department of Veterinary Microbiology, University of Saskatchewan, Saskatoon, SK, Canada

**Keywords:** *Salmonella*, biofilms, curli, cellulose, gastroenteritis, host adaptation

## Abstract

*Salmonella* are important pathogens worldwide and a predominant number of human infections are zoonotic in nature. The ability of strains to form biofilms, which is a multicellular behavior characterized by the aggregation of cells, is predicted to be a conserved strategy for increased persistence and survival. It may also contribute to the increasing number of infections caused by ingestion of contaminated fruits and vegetables. There is a correlation between biofilm formation and the ability of strains to colonize and replicate within the intestines of multiple host species. These strains predominantly cause localized gastroenteritis infections in humans. In contrast, there are salmonellae that cause systemic, disseminated infections in a select few host species; these “invasive” strains have a narrowed host range, and most are unable to form biofilms. This includes host-restricted *Salmonella* serovar Typhi, which are only able to infect humans, and atypical gastroenteritis strains associated with the opportunistic infection of immunocompromised patients. From the perspective of transmission, biofilm formation is advantageous for ensuring pathogen survival in the environment. However, from an infection point of view, biofilm formation may be an anti-virulence trait. We do not know if the capacity to form biofilms prevents a strain from accessing the systemic compartments within the host or if loss of the biofilm phenotype reflects a change in a strain’s interaction with the host. In this review, we examine the connections between biofilm formation, *Salmonella* disease states, degrees of host adaptation, and how this might relate to different transmission patterns. A better understanding of the dynamic lifecycle of *Salmonella* will allow us to reduce the burden of livestock and human infections caused by these important pathogens.

## Introduction

### *Salmonella* Nomenclature, Disease States, and Worldwide Impact of Infections

**Figure 1:**
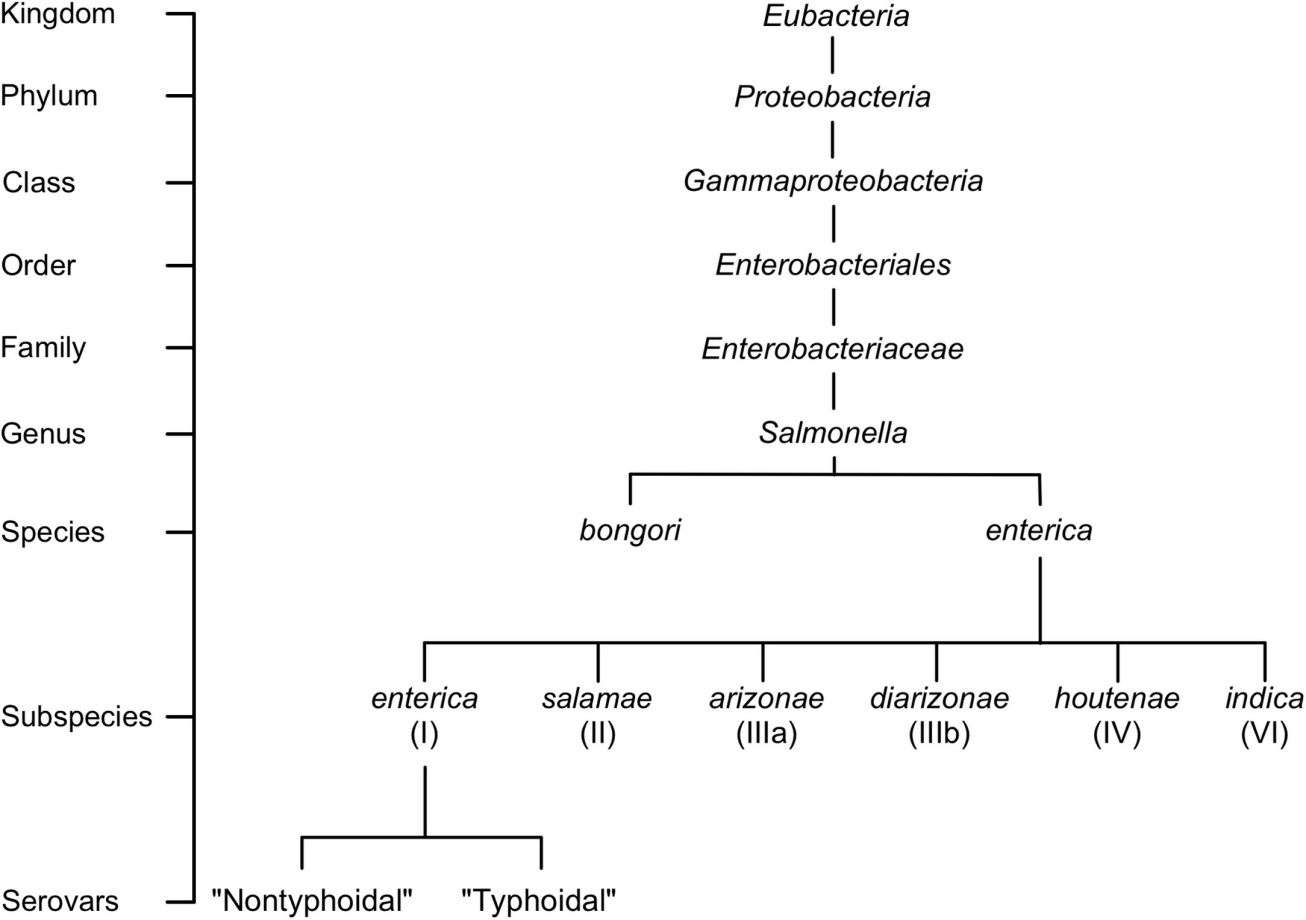
*Salmonella* taxonomy and general classifications. The genus *Salmonella* is classified into species, subspecies, and serovars based on the White–Kauffman–Le Minor scheme. Serovars are often grouped into non-typhoidal or typhoidal categories; however, this referencing approach is not a part of the official *Salmonella* classification scheme. For a expanded version of the taxonomical distribution of *Salmonella*, readers are referred to Ref. (9).

The current system of *Salmonella* nomenclature is based on layers of genetic, biochemical, and serological classification. New *Salmonella* strains can be categorized into species and subspecies according to DNA relatedness at the genomic level, originally shown through DNA–DNA hybridization, and the presence or absence of 11 biochemical traits (1, 2). Two species have been defined, *Salmonella bongori* and *Salmonella enterica*, of which *S. enterica* is further split into six subspecies that are designated by a Roman numeral and name (I, *enterica*; II, *salamae*; IIIa, *arizonae*; IIIb, *diarizonae*; IV, *houtenae*; and VI, *indica*) (Figure 1) (3). *Salmonella* are further subdivided into serovars using the classical Kauffman and White classification system (4). Serovars are representative of a unique combination of flagellar antigens (H1 and H2) and (lipopolysaccharide) oligosaccharide (O) or capsular polysaccharide (K) antigens (5). Using this classical system, more than 2,600 serovars have been identified, with their given name reflecting their combination of antigens, or in the case of serovars in subspecies *enterica*, a name representing their associated disease, host specificity, geographic origin, or relationship to other identified serovars (5, 6). Whole genome sequencing (WGS) is increasingly being used for classification, such as the sequencing of every *Salmonella* isolate by Public Health England (7) or the new typing scheme developed for *S. enterica* serovar Typhi (8). Strain typing *via* WGS has so far demonstrated promising results, both by supporting the current structure of *Salmonella* serovar nomenclature and by providing improved resolution of the phylogenetic relationship between *Salmonella* isolates (7). In regards to infection, subspecies I, or *S. enterica* subspecies *enterica* is the most well-represented among serovars and disease, accounting for approximately 60% of all serovars identified and greater than 95% of *Salmonella* isolates obtained from humans and domestic mammals (9, 10). In contrast, *Salmonella* isolates belonging to the remaining species and subspecies are normally obtained from cold-blooded hosts, and are only occasionally able to cause infections in humans (11).

Pathogenic *Salmonella* strains cause three main types of infections in humans. Gastroenteritis (150 million annual cases) is caused by many of the serovars in subspecies *enterica*, with serovars Typhimurium and Enteritidis being the most common (12, 13). In immunocompetent individuals, gastroenteritis infections involve the short-term colonization of the pathogen within the gastrointestinal (GI) tract, resulting in a localized inflammatory immune response accompanied by profuse diarrhea (14). The *Salmonella* serovars causing gastroenteritis are collectively referred to as non-typhoidal *Salmonella* (NTS). Enteric or typhoid fever (26.9 million annual cases) is caused by *S. enterica* serovars Typhi and Paratyphi, which are known as typhoidal *Salmonella* (15). This distinct disease involves invasion of the extra-intestinal compartment, often without the induction of inflammation or diarrhea (16). While such infections can occur asymptomatically, clinical manifestations of typhoidal *Salmonella* infections may include a persistent and gradual fever that elevates in a stepwise manner, as well as other symptoms, such as headache, chills, nausea, coughing, malaise, or a rapid pulse (16). The yearly mortality attributed to typhoidal *Salmonella* is estimated at ~145,000 deaths, which is more than double the number of deaths associated with gastroenteritis (15). After spreading systemically in their host, typhoidal *Salmonella* have the potential to persist for several weeks to years as a result of the pathogen’s intracellular association with monocytes and macrophages and potential long-term colonization of the gall bladder (17). The third human disease is caused by a group of NTS strains that cause systemic infections and have an increased association with bloodstream infections in sub-Saharan Africa (18). Like typhoidal *Salmonella* infections, invasive non-typhoidal *Salmonella* (iNTS) disease frequently lacks diarrheal symptoms, with febrile illness being the dominant clinical presentation in 95% of cases (19). This disease has a huge burden of mortality in the hardest hit areas, with an estimated 681,000 deaths per year. Due to non-specific symptomology and multidrug resistance of iNTS strains, there are often poor clinical outcomes despite correct diagnosis (18). A review of several clinical studies has revealed a significant association between invasive infections with NTS and immunocompromised populations, particularly children with malnutrition or severe malaria and adults with advanced infections of human immunodeficiency virus (HIV) (18–20). Failure of the immune system to maintain the intestinal epithelial barrier or to control intracellular *Salmonella* infections in these individuals provides a unique opportunity for NTS serovars to persist in the host (19).

*Salmonella* species continue to have a significant impact on global health. In a recent study analyzing the impact of 22 of the world’s most important foodborne pathogens, non-typhoidal, and typhoidal *Salmonella* were listed #1 and #2 in terms of disability adjusted life years (DALY) (15). The DALY metric is a measure of the overall disease burden, or the number of years lost due to ill-health, disability or early death. However, it does not take into account the economic impact of disease. In the United States of America alone, the impact of *Salmonella* infections related to hospital time, treatment costs, and lost work productivity have been estimated in the billions of dollars each year (21, 22). In addition to these estimates, *Salmonella* also presents as a significant economic challenge for producer groups and governments that are tasked with screening for and eliminating these pathogens within livestock species. Taken together, the different measures of impact demonstrate why it is so important to understand the complete *Salmonella* lifecycle, including how cells survive, persist and are transmitted between hosts.

## Biofilm Formation in *Salmonella*

The majority of bacterial life in nature is thought to exist in biofilms, a mode of growth where cells aggregate and become embedded in a self-produced extracellular matrix, usually in contact with a physical surface. Up to 40% of human and livestock diseases are thought to be biofilm-related and have enormous medical and economic impacts (23, 24). In addition, most of these biofilms are polymicrobial in nature (25). The exact reasons why bacteria aggregate together are not fully understood. There are examples of emergent behaviors associated with aggregation of larger numbers of cells, such as the enhanced breakdown of chitin by *Vibrio cholerae* (26). There is also the possibility that polymer production within the biofilm is the result of cells competing with each other for access to oxygen or nutrients (27). The presence of extracellular polymers themselves can create a unique microenvironment for cells within a biofilm, by inducing potential oxygen gradients (28, 29), or signaling nutrient limitation (30). An example of this was demonstrated for *Pseudomonas aeruginosa*, where the presence of DNA in the extracellular matrix imposed a cation restriction on the cells inside the biofilm, which in turn led to increased antibiotic resistance in the biofilm cells (31). Thus, the characteristic properties of a biofilm may be due in part to the physical barriers provided by the matrix polymers and also to the microenvironments induced by growth at high cell densities within the matrix.

### General Description of *Salmonella* Biofilm Types

**Figure 2:**
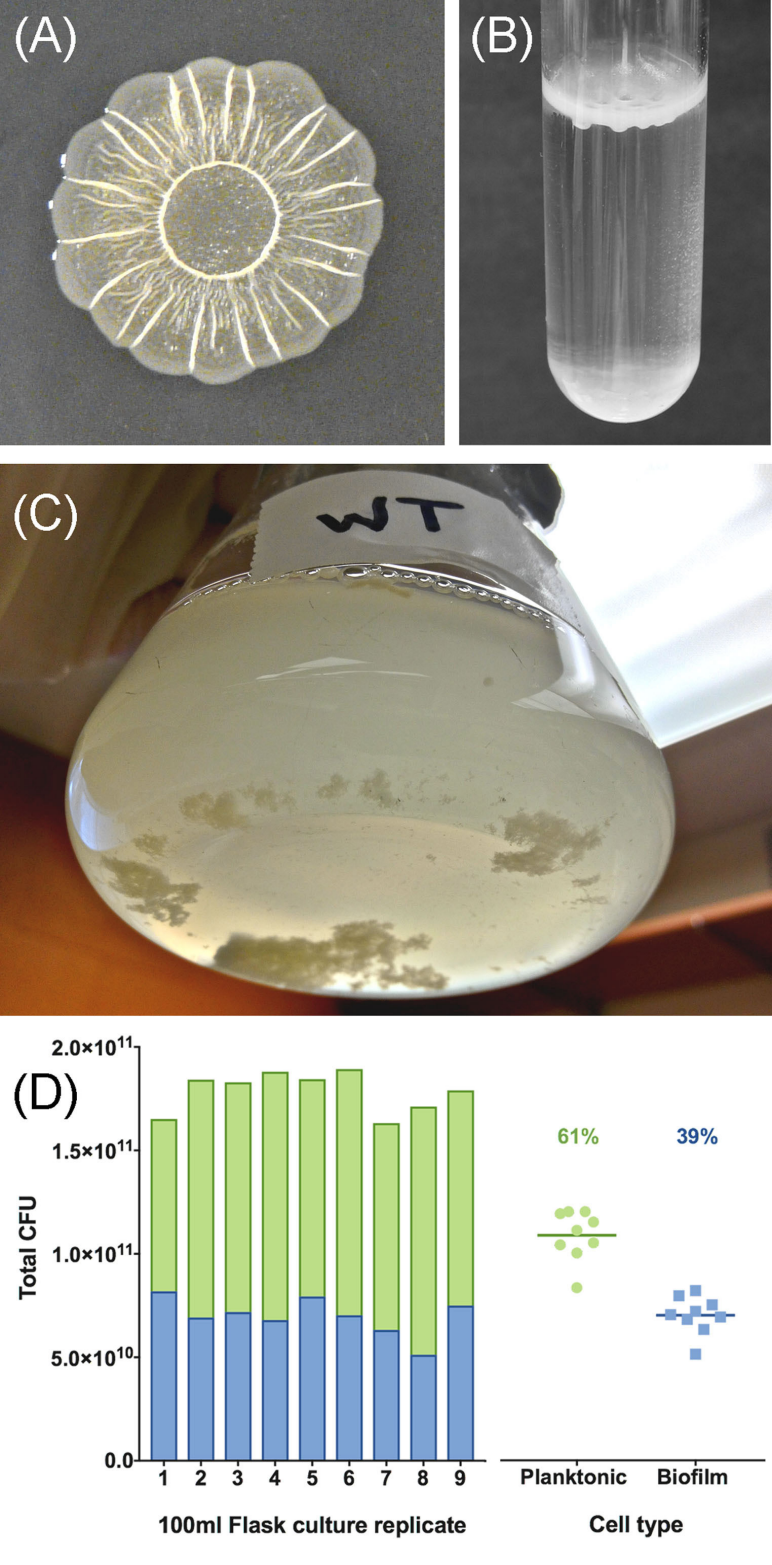
Examples of *Salmonella* biofilm formation. **(A)** Colonies grown for 48 h at 28°C on solid 1% tryptone media form the characteristic surface patterns of the *r*ed, *d*ry, *a*nd *r*ough (rdar) morphotype. The colony appears red when the media is supplemented with the dye Congo red. **(B)** Pellicle formation at the air–liquid interface of a 1% tryptone liquid culture [adapted from Ref. (46)]. **(C)** *Salmonella* form multicellular aggregates and planktonic cells within the bulk liquid phase of a flask culture. **(D)** The number of colony forming units (CFU) present in aggregate or planktonic cell subpopulations from **(C)** was calculated using conversion factors determined from serial dilution plating after homogenization (1.92 × 10^9^ CFU per 1.0 OD_600_ for planktonic cells; 1.73 × 10^8^ CFU/mg for aggregates). The green bars and blue bars represent the proportion of planktonic cells and aggregates comprising the total number of cells in the population; points on the right side of the graph represent total CFU values for each cell type from nine replicate flask cultures. The percentage values represent the average proportion of each cell type.

For *Salmonella*, the best studied biofilm phenotype has been termed the rdar morphotype, named for the *r*ed, *d*ry, *a*nd rough appearance of colonies grown on agar plates containing Congo red dye (32, 33). Congo red accumulates within the rdar colony due to the presence of the proteinaceous curli fimbriae, which are functional amyloid structures that are resistant to detergents, pH, and proteases (32, 34), and cellulose, the β1-4-linked glucose polymer, which is another resistant polymer (35). These two components function as the extracellular matrix scaffold, with curli providing short-range interactions between cells and cellulose providing long-range interactions over the distance of the entire colony (36). Together their production leads to a rough and dry colony appearance (37) (Figure 2A). There are other polymers known to be present within the rdar extracellular matrix, such as the O-antigen capsule (38) and polysaccharides yet to be fully characterized (39), as well as proteins, such as flagella, that contribute to the architecture of the resulting colony (40). In standing liquid cultures, biofilms with a matrix comprised of curli and cellulose have been described as pellicles, which refer to the film of cell growth that appears at the air–liquid interface (33, 41, 42) (Figure 2B). BapA, a large *Salmonella* protein containing numerous repeated sequences, has been shown to contribute to the strength and integrity of these pellicles (43). There are also biofilms formed at the air–liquid interface in severely nutrient-limited liquid media that are composed of cellulose, but not curli (42, 44). We recently developed an *in vitro* flask model for studying *Salmonella* biofilm development, where the cells in the culture differentiate into two distinct populations: multicellular aggregates and planktonic cells. The multicellular aggregates produce the same polymers as standard biofilms (45) and accumulate in the bottom of the flask, whereas the planktonic cells remain suspended in the growth media (Figure 2C). The proportion of each cell type within replicate flask populations is relatively stable (Figure 2D).

**Figure 3:**
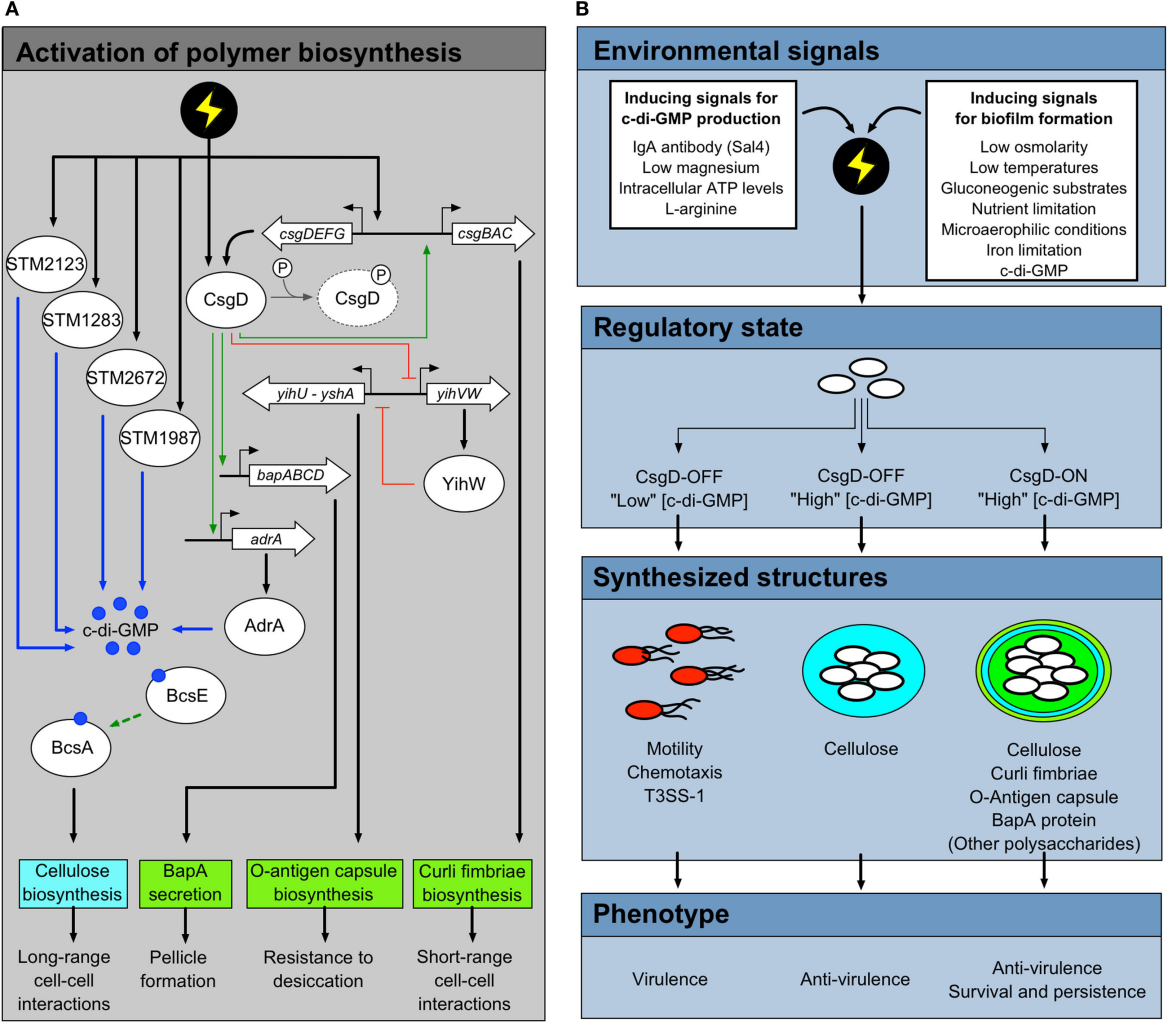
*Salmonella* phenotypes that result from the activity of CsgD and c-di-GMP. **(A)** Synthesis of biofilm-associated polymers is regulated at the genetic level by CsgD and the secondary messenger molecule c-di-GMP. Multiple environmental conditions act as inducing signals for *csgD* expression, CsgD synthesis, and c-di-GMP production. These environmental conditions (represented here as a lightning bolt) are transduced into intracellular signals *via* outer membrane proteins, two-component signal transduction systems, regulatory proteins, and enzymes associated with c-di-GMP production. A select set of diguanylate cyclases (STM1283, STM2123, STM2672, and STM1987) can contribute to the c-di-GMP pool that induces BcsE and BcsA activity, resulting in cellulose biosynthesis. CsgD is the master transcriptional regulator associated with *Salmonella* biofilm formation. In its unphosphorylated active state, CsgD promotes the expression of *adrA*, a potent diguanylate cyclase associated with promoting cellulose biosynthesis. CsgD is additionally responsible for activating the transcription of genes and operons associated with the biosynthesis of curli fimbriae, O-antigen capsule, and BapA protein. Genes and operons are shown as open arrows, proteins as ovals, and c-di-GMP molecules as dark blue circles. Positive regulation is denoted as green arrows, while regulatory inhibition is shown as flat-headed red arrows. Activation *via* c-di-GMP molecules is shown as blue arrows. **(B)** Multiple environmental signals can induce the biosynthesis of biofilm polymers. However, some conditions can activate c-di-GMP production and cellulose biosynthesis independently from other biofilm polymers. Under biofilm-inducing conditions, only a subset of *Salmonella* cells in the total population will synthesize high levels of CsgD. This subpopulation enters a CsgD-ON state, which results in significant c-di-GMP production and biosynthesis of biofilm matrix polymers. Cells within the biofilm are able to survive and persist in harsh environmental conditions. In contrast, some *Salmonella* cells in the population do not have sufficient synthesis of CsgD, resulting in a CsgD-OFF state and subsequently low intracellular concentrations of c-di-GMP. These cells remain in a planktonic state, are highly motile, and synthesize the type-three secretion system (T3SS)-1, resulting in a virulent cell subpopulation. Due to the CsgD-independent activity of some diguanylate cyclases, *Salmonella* cells can have high intracellular levels of c-di-GMP while in a CsgD-OFF state. As such, these cells may synthesize cellulose in the absence of other major biofilm matrix polymers. The relatively low expression/activity of virulence-associated factors in cells aggregated together within cellulose or other biofilm polymers is due at least in part to the state of c-di-GMP pools within the cells.

Each of the biofilms described above, except for cellulose-dominated biofilms formed on glass, are related in regulatory mechanisms and are activated in a similar way (Figure 3A). Regulation feeds through CsgD, a transcriptional regulatory protein that activates the biosynthesis of the majority of biofilm polymers described above (46, 47). The favored growth conditions for biofilm formation are media of low osmolarity, at temperatures below 30°C, and in the presence of gluconeogenic substrates, such as amino acids (33). Nutrient limitation is known to activate polymer production, but there are many inputs into the *csgD* promoter, which is part of one of the most complex regulatory networks in *Salmonella* [see Ref. (47) or (48) for a comprehensive review]. Important biofilm-activating factors include microaerophilic oxygen levels (49), iron limitation (33) and the presence of bis-(3′–5′)-cyclic dimeric guanosine monophosphate, or cyclic-di-GMP (50). Cyclic-di-GMP is a bacteria-specific secondary messenger molecule that is known to activate biofilm formation when produced at high levels, almost universally in all bacterial species where it has been examined [see Ref. (51) for a review]. In *Salmonella*, there are 22 enzymes that can potentially regulate c-di-GMP levels, but only a subset affect *csgD* expression directly (44, 52). Finally, the presence of glucose has been shown to repress biofilm formation and *csgD* transcription (53, 54). This indicates that biofilm formation is a choice heavily influenced by the growth potential in the environment surrounding the cells.

*S. enterica* serovar Typhi has been shown to form unique biofilms on the surface of gallstones (55, 56). These biofilm-coated gallstones have been observed in association with chronic human Typhi carriers (57) and the presence of bile is a key inducing factor (56). Curli fimbriae are not involved in the formation of these biofilms, although the O-antigen capsule and flagella have been implicated (58, 59). These biofilms are distinct from the rdar morphotype and are specific to serovar Typhi; they are likely to have a unique function in the *Salmonella* lifecycle and as such, are not discussed extensively in this review.

### Biofilm as a Survival Advantage

In the early days of genome-wide comparisons in *Salmonella*, researchers had identified multiple different fimbrial types, each with scattered distribution between the different *S. enterica* serovars (60). For the current picture of fimbrial distribution, see Ref. (61, 62). Due to pioneering work with Type I and P fimbriae in *E. coli* (63, 64), it was predicted that the presence of different fimbrial types would allow *Salmonella* cells to attach to the intestinal epithelium of specific host species (65). One example was the association of SEF14 fimbriae with poultry-associated *S. enterica* serovars (60, 66). Curli fimbriae were unique in that they were conserved in the *Salmonella* genus (60); the *csgA* gene coding for the major curli subunit (formerly *agfA*) was developed as an early *Salmonella* diagnostic, detectable in 603 of 604 tested strains (67). Curli fimbriae and the corresponding biosynthetic operons were also detected in *E. coli* (60, 68, 69), which had a last common ancestor with *Salmonella* approximately 100 million years ago (70). Hammar et al. identified the presence of two polycistronic *csg* operons responsible for curli biosynthesis in *E. coli* (71), which were later identified in *Salmonella* (72) and subsequently shown to be interchangeable cross-species (69). These findings brought up interesting questions about curli fimbriae and why they would be conserved in *Salmonella* strains that were capable of colonizing so many different host species. It indicated that curli fimbriae were involved in a common aspect of the *Salmonella* lifecycle that was shared by many diverse strains.

One clue about curli function came as a result of the unusual protocol for curli purification. After the majority of *S. enterica* serovar Enteritidis cellular material was solubilized or had been removed by enzymatic digestion, purified curli were isolated from the top of a preparative SDS-PAGE, as the material that did not enter the gel (32). Purified curli fibers remained intact after boiling in the presence of SDS, exposure to sodium hydroxide, or digestion with proteinase K, treatments that would depolymerize or degrade most if not all other fimbrial types (32). Resuspension in >70% formic acid was the only treatment found to depolymerize the curli fibers into their structural subunits (32, 73); it should be noted that no improved alternative to this procedure has been published in the last 25 years. We know now that curli fimbriae are a functional amyloid with extensive cross-β structure (74, 75), hence their extreme stability. It was apparent early on that curli fimbriae could provide physical stability and possible resistance for aggregated *Salmonella* cells.

Another indication of the potential function of curli fibers came from the optimal growth conditions for their production *in vitro*. Curli fimbriae were originally discovered within a strain of *E. coli* isolated from cattle manure (76). The fimbriae originally discovered in *S. enterica* serovar Enteritidis by Collinson et al. were thought to be distinct from curli fimbriae in *E. coli* due to differences in amino acid residues and due to constitutive expression in serovar Enteritidis at 37°C and 28°C (32). However, the amino acid differences were based on the false identification of the curli subunit by Olsén et al. (76), who had in fact identified Crl, a biofilm transcriptional regulatory protein (named Crl for curli). The constitutive production of curli in serovar Enteritidis was found to be due to a single nucleotide polymorphism (SNP) in the promoter region that changed the transcriptional regulation (33). Most strains of *Salmonella*, it has since been found, only produce curli fimbriae at temperatures below 32°C (76). In addition, curli production requires low osmolarity and appears to be triggered by nutrient limitation (32, 33, 77). One could envision that similar conditions might naturally exist in non-host environments. Hence, researchers started to think more about curli production and *Salmonella* aggregation in the context of the environment.

Anriany et al. (78) first analyzed the survival properties of rdar colonies formed by *Salmonella* serovar Typhimurium (78), due to the apparent similarities to the “rugose” colony morphology in *V. cholerae*, which had previously been shown to enhance *Vibrio* survival (79). Cells in rdar colonies had enhanced survival upon exposure to hydrogen peroxide and to acidic pH, as compared to non-rdar colonies. Cellulose was discovered as the second major component of the *Salmonella* biofilm extracellular matrix (42, 80); it is also extremely resistant and forms extensively hydrogen-bonded sheets (35). Solano et al. (42) treated rdar colonies with sodium hypochlorite, which is used as a common waterline disinfectant, and showed that the presence of cellulose provided protection to the cells. Other researchers extended these results to include pellicle biofilms formed at the air–liquid interface in liquid cultures (81). Scher et al. also tested heat and acidity but the pellicle cells were not significantly more resistant than stationary-phase cells. We performed a series of survival experiments, taking advantage of the unique properties of rdar colonies, specifically that they can be lifted off the agar surface in one piece (33). We started placing intact colonies on plastic surfaces and allowing them to dry out before periodically inoculating pieces of these colonies into fresh liquid media. Within 1–2 months, we realized that not only were cells staying viable in this dried out state, but also there was no apparent lag-time when placed into fresh media. Therefore, we performed an experiment comparing survival of rdar colonies to colonies formed by *Salmonella* mutant strains without curli, without cellulose, or without the entire extracellular matrix (Δ*csgD*) (37). After 3 months, the rdar colonies displayed 3–10 times enhanced survival compared to the biofilm mutants. After 9 months, the difference was as high as 30 times increased survival and exposure of the dried colonies to sodium hypochlorite yielded an even bigger survival difference. At the time that these desiccation experiments were performed, collaborators had discovered a new polysaccharide capsule in *Salmonella* that was part of the biofilm extracellular matrix (38). Gibson et al. performed desiccation experiments using a lyophilizer, and proved that the O-antigen capsule was the major factor providing desiccation resistance to cells. This reinforced the role of polysaccharides in the biofilm matrix to maximize water retention, nutrient trapping, and provide buffering (82).

As a final test of the potential importance of the biofilm extracellular matrix in the survival of *Salmonella* cells, we examined the viability of dried out rdar morphotype colonies after 2.5 years (83). The recovery of these cells was problematic in that they did not grow well on a variety of selective media commonly used for *Salmonella* isolation, such as SS or XLD agar. On non-selective media, the recovery rate after 30 months was ~5% of the starting number of cells; however, when evaluating viability using a live-dead cell stain, over 50% of cells appeared to be alive (84). The discrepancy in measured cell number between plating and live-dead staining suggested that cells might be in a type of viable, non-culturable (VBNC) state (85, 86). The existence of a VBNC state would add to the difficulty in eradicating *Salmonella* in agricultural or food-processing settings. Perhaps most importantly, cells in 2.5-year old rdar colonies retained an ability to cause infections in the mouse model of infection (83). There are many differing theories about how *Salmonella* can persist in industrial and/or agricultural settings, including survival in the local rodent or insect populations (87–89). The survival results for rdar colonies indicate that *S. enterica* strains could survive on their own in dryness or without exogenous nutrients for a long period of time.

In agricultural and industrial settings, biofilm formation has long been considered a factor to explain the extreme persistence of *Salmonella*. Outbreaks of human gastroenteritis have been linked to the consumption of a wide variety of foods or food products, not just fresh foods that may come into contact with contaminated water sources but also processed foods that go through extremes of dryness (84). Often researchers have analyzed the isolates/strains that are associated with outbreaks or with agricultural/industrial persistence to check their biofilm-forming ability, which has tended to be overwhelmingly positive (90–92). However, as yet, there has been no positive confirmation that the rdar morphotype is “the” critical factor. We performed a project examining *Salmonella* colonization of egg-conveyer belts used in modern poultry barns, because one farm had their flock re-infected by the same strain of *S. enterica* serovar Enteritidis over a 3-year period and the conveyor belt was identified as the source of contamination. Isolates taken from each year all formed rdar colonies, the rdar colonies were resistant to treatment with common disinfectants used in the industry, and all isolates formed robust biofilms on pieces of egg belt (93). However, the presence of rdar biofilm had no measurable effect on survival when contaminated pieces of egg belt were treated with disinfectant. We concluded that it is difficult to recreate real-world situations in an *in vitro* setting.

Several studies have provided convincing evidence to implicate biofilm formation as an important factor in the interaction between *Salmonella* and plants [reviewed in Ref. (94)]. Curli fimbriae, cellulose, and O-antigen capsule are involved in different stages of plant colonization and *Salmonella* persistence within or on plant tissue (95–97). In their laboratory study, Lapidot and Yaron reported the observation of multicellular aggregates beneath the surface of parsley leaves grown in soil irrigated with contaminated water (98). Perhaps, the closest real-world evidence of enhanced biofilm formation having a biological impact was with the recent *E. coli* outbreak in Germany in 2011. The O104:H4 strain that was associated with contaminated fenugreek seeds caused the highest rates of hemolytic–uremic syndrome ever recorded and displayed clinical evidence of increased biofilm formation (99). Analysis of this strain showed that it had acquired a unique gene that led to enhanced biofilm formation (100). It is possible that biofilms were involved in the initial attachment to the fenugreek seeds, as previously demonstrated for alfalfa seeds (101), which would have facilitated both the dissemination and persistence of the outbreak strain.

### Biofilm as an Anti-Virulence Trait

Collinson et al. first proposed that aggregation could provide *Salmonella* with a mechanism for surviving the harsh conditions of the host intestinal tract to ensure that a “viable and sufficient” inoculum could reach the epithelial layer (32). This seemed a valid hypothesis because although the dogma was that a high infectious dose was required for *Salmonella* infections, there were multiple epidemiological trace-backs to outbreaks that seemingly had a low inoculum (102). One could envision small aggregates being taken up by a host and the presence of resistant extracellular matrix polymers shielding cells during passage through the stomach. This would lower the infectious dose required to cause infections. The biofilm flask cultures described (Figure 1), provided an opportunity to test this hypothesis. Cultures of wild-type *S. enterica* serovar Typhimurium were competed in mouse infections with an isogenic, curli-negative mutant that was unable to aggregate. To our surprise, the non-aggregated mutant strain consistently outcompeted the wild-type strain (45). In our more recent studies, planktonic and aggregate cell types were isolated from the biofilm flask cultures, and competed during co-infections of mice. Again, the non-aggregated cells consistently outcompeted the aggregates (103). There is other recently published evidence that the presence of extracellular matrix factors reduce *Salmonella* virulence in the mouse model of infection (104). The results from these studies have established that the formation of rdar biofilms is not a virulence adaptation.

The results above created a conundrum because curli fimbriae have been well established as potent inducers of the innate immune system. Curli represent a pathogen-associated molecular pattern (PAMP) that causes activation of toll-like receptors 1 and 2 (105, 106), as well as intracellular NOD-like receptors (107). There is also evidence that curli can bind to multiple host proteins, such as contact-phase proteins or extracellular matrix proteins fibronectin and laminin [reviewed in Ref. (34)]. Although this research would seem to indicate that curli are produced *in vivo*, studies have yet to show that CsgD and curli are produced during infection. Deletion of *csgBA* (formerly *agfBA*), encoding the main curli subunit proteins, caused no noticeable impairment of *Salmonella* virulence (108). We monitored the expression of a curli reporter fusion *in vivo* using a whole animal imager, but we did not detect expression in any of the 13 mice that were screened (45). We hypothesized that the host immune system does interact with *Salmonella* rdar biofilms during an infection, perhaps immediately after ingestion of *Salmonella* biofilm cells.

Cellulose, the other main structural component of rdar biofilms, may have an important role in host–pathogen interactions during *Salmonella* infection. In contrast to curli biosynthesis, which is regulated at the transcriptional level by CsgD (69, 72), the *bcs* operons encoding the cellulose biosynthesis enzymes are transcriptionally independent of CsgD and the rdar morphotype (80). Activation of cellulose production is largely dependent on allosteric activation of the BcsA subunit by c-di-GMP (51). CsgD is responsible for inducing the expression of *adrA*, the first diguanylate cyclase enzyme to be associated with cellulose biosynthesis in *Salmonella* (36, 50). The transcriptional uncoupling of cellulose biosynthesis from CsgD regulation thus provides an opportunity for cellulose production to occur independently of the biofilm phenotype. As stated above, the *Salmonella* genome contains multiple diguanylate cyclase genes. The evidence for a CsgD-independent pathway for cellulose production was first provided by Ref. (42) and has since been demonstrated in *E. coli* (109). In a pair of subsequent studies, Lasa and colleagues performed systematic deletion and re-integration of the genes for each c-di-GMP synthase enzyme, resulting in the identification of four c-di-GMP synthesizing enzymes that can each independently induce *Salmonella* biofilm formation at 37°C (44, 110) (Figure 3A).

Within the host, there are potentially multiple cues that can induce c-di-GMP-based activation of cellulose biosynthesis. Co-incubation of *Salmonella* with Sal4, a protective IgA antibody secreted into the intestinal lumen, has been shown to increase c-di-GMP levels and cellulose production through activation of YeaJ (STM1283) (111), one of the enzymes identified by Lasa and colleagues. Cellulose has also proven important for attachment of *E. coli* and *Salmonella* to the surface of intestinal cells, although the exact c-di-GMP synthase enzymes responsible for this process are still unknown (112, 113). Further, *Salmonella* have been shown to produce measurable amounts of cyclic-di-GMP and cellulose while inside macrophages in response to low intracellular concentrations of magnesium and to changes in intracellular ATP levels (114). These authors discovered the production of cellulose *in vivo* by studying a *mgtC* mutant strain. *mgtC* is a key virulence gene that is expressed inside macrophages; deletion of *mgtC* caused attenuation of virulence and an increase in cellulose production. However, the *mgtC* mutant strain was no longer attenuated if the accumulation of cellulose was prevented, suggesting that cellulose was impeding virulence. Most surprisingly, these authors found that a *Salmonella* cellulose synthase mutant was hypervirulent, and killed mice faster than the wild-type strain (114). Ahmad et al. also recently published evidence that cellulose impedes *Salmonella* virulence; synthesis of BcsZ was necessary to reduce cellulose production *in vivo* and to maintain virulence (115). Mills et al. (116) identified l-arginine as another potential signal that is present inside the macrophage and is able to induce c-di-GMP biosynthesis and cellulose production. Finally, previous work out of the Romling lab demonstrated that cellulose production inhibits host cell invasion (117, 118). Altogether, the evidence indicates that *Salmonella* cellulose production plays a key role in host–pathogen interactions and that the interactions may be much more complex than initial experiments demonstrating that cellulose production was not essential for *Salmonella* virulence (42).

## Correlation Between Biofilm Formation and Host Specificity

Since the discovery of curli fimbriae and description of the rdar morphotype, researchers have examined the conservation of this phenotype within the *Salmonella* genus. These data are summarized in Table 1. In general, the rdar morphotype is conserved in a majority of non-typhoidal *S. enterica* serovars, such as Enteritidis and Typhimurium, in strains that normally would cause gastroenteritis. We proved that *csgD* (curli) promoter function was conserved in a diverse collection of strains, representing *S. bongori* and all six *S. enterica* subspecies (119). However, many of the strains had lost biofilm formation due to *trans* changes in the rdar regulatory network likely caused by domestication of the strains (46, 120). This provided a cautionary note that these kinds of extracellular phenotypes can be easily lost during laboratory passage (120).

**Table 1:** Biofilm formation among host-generalist and host-adapted *Salmonella* strains.

Study	*Salmonella enterica* serovars (# strains)	Biofilm formation^a^	Strain origins	Special notes^b^
Solano et al. (42)	Enteritidis (204 strains)	40 of 56 (71%)	Animal	Overall biofilm formation 198 of 204 (97%)
		48 of 63 (76%)	Clinical	
		27 of 54 (50%)	Environmental	
		20 of 31 (65%)	Food	

Römling et al. (121)	Typhimurium, Enteritidis (>800)	720 of ~800 (90%)	Animal, human	Collected at National Reference Center; Germany

Solomon et al. (122)	28 serovars (71 strains)^c^	11 of 15 (73%)	Clinical	Overall curli production was 93%
		26 of 31 (84%)	Meat	
		14 of 25 (56%)	Produce	

White et al. (37)	37 serovars (72 strains)	58 of 72 (80.5%)	*Salmonella* reference collection B (SARB);	Boyd et al. (123)

Malcova et al. (124)	Typhimurium (84)	76 of 84 (90%)	Animal species	Collected 2004–2007 in Czech Republic

Vestby et al. (92)	Agona (47), Montevideo (38), Senftenberg (42), Typhimurium (21)	110 of 148 (74%)	Clinical and feed and fish meal factories in Norway	Overall curli production 100%; 55% Agona and Seftenberg isolates were cellulose-negative

De Oliveira et al. (90)	Serovars undetermined (174)^d^	96 of 174 (55%)	Raw poultry isolates from Brazil	Overall biofilm formation 171 of 174 (98%)

Laviniki et al. (91)	16 serovars (54 strains)^e^	54 of 54 (100%)	Ingredients, equipment—4 feed mills in Brazil	

Ramachandran et al. (125)	Typhimurium ST19	5 of 5 (100%)	Human blood isolates in Mali + one reference	Gastroenteritis-causing isolates

White and Surette (46)	Subspecies I, II, IIIa, IIIb, IV, VI, and *S. bongori* (group V)		*Salmonella* reference collection C	Boyd et al. (119); IIIa strains had inactivating single nucleotide polymorphisms (SNPs) in *csgD* promoter (curli); signs of domestication in lab collection
	Lab collection	5 of 16 (31%)		
	Remaining strains	72 of 80 (90%)		

Römling et al. (121)	Typhi	0 of 19 (0%)	Human	Several Gallinarum strains were cellulose positive at 37°C
	Choleraesuis	0 of 17 (0%)	Animal, human	
	Typhimurium v. Copenhagen	0 of ~80 (0%)	Pigeon	
	Gallinarum	1 of 23 (4%)	Animal, human	

Malcova et al. (124)	Typhimurium v. Copenhagen	0 of 10 (0%)	Pigeon, duck	These are phage type DT2 isolates

White et al. (45)	Typhi	0% of >200 isolates	Human	*Salmonella* genetic stock center

Singletary et al. (126)	Typhimurium ST313		Lineages referred to in Okoro et al. (127)	Common SNP in *bcsG* (cellulose) identified in lineage II isolates, including the type strain, D23580
	African Lineage I	3 of 3 (100%)		
	African Lineage II	0 of 6 (0%)		

Ramachandran et al. (125)	Typhimurium ST313	0 of 11 (0%)	Human blood isolates from Mali + reference strains	Authors suggested that ST313 isolates may not be able to persist in the environment
	Typhi	0 of 6 (0%)		
	Paratyphi A	0 of 3 (0%)		

Ashton et al. (128)	Typhimurium ST313	0 of 16 (0%)	Human clinical isolates from UK	Genome degradation in African lineage II strain D23580 conserved in UK isolates
	African Lineage II			

*^a^Most studies have tested strains for ability to form *r*ed, *d*ry *a*nd *r*ough (rdar) colonies on media containing Congo red; this indicates curli and cellulose production. Biofilm-negative strains were *s*mooth *a*nd *w*hite (saw) on this media. It is typical for isolates to be rdar at 28°C and saw at 37°C. In some cases, researchers grew strains on media containing calcofluor and tested for fluorescence as confirmation of cellulose production*.

*^b^Overall biofilm results are reported in cases where researchers have tested strains in a variety of growth conditions*.

*^c^Serovars Anatum (2), Baildon (1), Branderup (1), Bredeney (1), Derby (1), Enteritidis (5), Hadar (3), Gaminara (2), Heidelberg (3), Hidalgo (1), Infantis (1), Kentucky (2), Mbandaka (2), Michigan (1), Montevideo (2), Muenchen (1), Muenster (2), Newington (1), Newport (3), Oranienburg (1), Poona (6), Reading (1), Saint Paul (3), Saphra (1), Schwarzengrund (2), Stanley (1), Thompson (3), Typhimurium (13), Worthington (1)*.

*^d^Most commonly isolated Salmonella strains from poultry in Brazil are from serovar Enteritidis*.

*^e^Serovars Agona (5), Anatum (4), Cerro (1), Infantis (2), Mbandaka (1), Montevideo (18), Morehead (1), Newport (2), Orion (3), O:3,10 (2), O:16:c:- (1), Schwarzengrud (1), Senftenberg (6), Tennessee (4), Typhimurium (1), Worthington (2)*.

Römling and colleagues first identified a connection between increased host adaptation in *Salmonella* strains and an inability to form biofilms (121). One particular variant of serovar Typhimurium (i.e., var. Copenhagen) that causes a systemic disease in pigeons (129) had lost the ability to form the rdar morphotype. Their analysis was expanded to include other host-adapted serovars, such as Cholerasuis (pigs), Gallinarum (chickens), and host-restricted serovar Typhi (humans), which were almost entirely rdar negative (Table 1). We reported that two Typhi strains were rdar positive (37); however, this was a mistake caused by the presence of a contaminating rdar-positive isolate within the stock cultures. We subsequently tested >200 Typhi isolates and all were rdar negative (45). We also identified *S. enterica* subspecies *arizonae* (IIIa) strains as being rdar negative due to inactivating SNPs in the *csgD* (curli) promoters (46). Subspecies *arizonae* isolates rarely cause human infections, but are frequently isolated from the gut of reptiles and snakes, and therefore, may be part of the commensal microflora (130). In *S*. Typhimurium var. Copenhagen isolates, the prevalent mutation was a G to T transversion in the −35 region in the *csgD* promoter, which may partially explain the loss of the biofilm phenotype (121). For *S*. Typhi, the intergenic region between the divergent curli operons has conserved sequence, but multiple mutations exist within the curli (*csg*) and cellulose (*bcs*) biosynthesis operons, where preliminary stop codons within *csgD* and *bcsC* genes may eliminate the possibility for synthesis of curli fimbriae and cellulose altogether (121, 131). We speculated that sequence mutations or *cis* changes in the *csg* genes/promoters are indicative of a change in the lifestyle of *Salmonella* isolates (46). Each of the serovars above that are lacking rdar biofilm formation have evidence of a restricted host range.

The most recent screening efforts have focused on iNTS strains from sub-Saharan Africa. With a few exceptions, it appears that biofilm formation is impaired in these strains (Table 1). Ramachandran et al. (125) performed colony desiccation and sodium hypochlorite experiments with serovar Typhimurium ST313 isolates and demonstrated that they were also impaired for survival. This preliminary evidence suggests that iNTS isolates may have undergone an evolutionary change in lifestyle when compared to their gastroenteritis-causing NTS counterparts.

## Strategies of *In Vivo* Infection

Central to *Salmonella* pathogenesis is its ability to modify host cell biology *via* two type-three secretion systems (T3SS), T3SS-1 and T3SS-2 (132). These specialized organelles span the bacterial inner and outer membranes and allow for the delivery of effector proteins into the cytoplasm of eukaryotic cells (133, 134). Type-three secretion systems are found exclusively in Gram-negative bacteria (135, 136). For *Salmonella*, genes for the T3SS-1 or T3SS-2 apparatus, regulatory components, and nearly all associated effector proteins are found within horizontally acquired DNA regions known as *Salmonella* Pathogenicity Islands (SPIs) (134). The SPI-1 region contains genes associated with T3SS-1, is found in all serovars of *S. enterica* and *S. bongori*, and is important for the invasion of intestinal epithelial cells (137, 138). In contrast, the full-length SPI-2 region harboring genes for T3SS-2 is present exclusively in *S. enterica*, and is associated with the intracellular survival of *Salmonella* within eukaryotic cells (137).

### Non-typhoidal versus Typhoidal *Salmonella*

Non-typhoidal *Salmonella* infections in immunocompetent individuals can be generalized into three important steps: invasion, inflammation, and intestinal replication. While decades of literature have been dedicated to understanding invasion and inflammation, recent research has uncovered important aspects of *Salmonella* replication and transmission in the inflamed intestine. In this section, we evaluate what is known about these important steps in gastroenteritis associated with NTS infections and consider how biofilm biology and *Salmonella* transmission may relate to our current understanding of *Salmonella* pathogenesis.

Following *Salmonella* entry into the host *via* contaminated food or water, cells travel through the digestive system and localize to the distal ileum and colon of the GI tract (139). *Salmonella* cells rely on flagellar motility and chemotaxis systems to traverse the intestinal mucus layer and identify sites on the host cell that are permissive for invasion (140). Fimbriae and other protein adhesins on the bacterial cell surface initiate association of the pathogen to the targeted epithelial cell; the needle complex of the T3SS-1 is critical for stabilizing this interaction. It is currently hypothesized that expression and synthesis of the T3SS-1 is induced by several important cues provided by the local host environment, including low-oxygen tension, high osmolarity, near-neutral pH, and acetate production levels from the resident microflora (141). The secretion apparatus is established in a step-wise manner, requiring formation of the basal body within the bacterial cell membranes to facilitate secretion of the needle complex (142). Proteins attached to the end of the T3SS-1 needle, collectively referred to as the translocon, are then inserted into the host cell membrane, creating a pore that allows for the injection of *Salmonella* effector proteins into the host cell (143). Establishment of a secretion-competent T3SS-1 is a mandatory prerequisite for the process of host cell invasion [reviewed in Ref. (144)]. Pathogen cells that traverse the epithelial cell layer encounter tissue mononuclear cells (i.e., macrophages and dendritic cells) within the lamina propria, resulting in the uptake of the pathogen into a phagosome (139). *Salmonella* depend on the T3SS-2 and associated effectors to manipulate the phagosome environment and promote pathogen survival and replication within the *Salmonella*-containing vacuole (SCV). The effectors are responsible for re-modeling the SCV environment and are hypothesized to provide a potential source of nutrients during pathogen replication (144).

Detection of PAMPs and components injected by the pathogen during its uptake into host cells elicits the production of a proinflammatory immune response (139). This response inhibits the spread of NTS past the lamina propria in three ways: (1) by activating infected macrophages and inducing killing of intracellular *Salmonella*, (2) through recruitment of neutrophils to the infection site for extracellular killing, and (3) by stimulating epithelial cells to release antimicrobial peptides into the intestinal lumen to control the replication of NTS cells (139). While host inflammation effectively controls the NTS cells that have invaded the epithelial cell layer, it acts as a potent stimulator of growth of the NTS population in the intestinal lumen (139). Recent studies have shown two mechanisms by which NTS are able to exploit the host inflammatory response. Epithelial cells release the antimicrobial agent lipocalin-2, a molecule that binds to enterochelin, an iron chelation molecule used by Gram-negative bacteria in the gut to acquire iron from the host (145). In addition to enterochelin, NTS are able to produce a second iron chelation molecule, salmochelin, which cannot be bound by lipocalin-2 (145). As a result, NTS cells in the intestinal lumen continue to replicate while the local microbiota are starved for iron. The low-oxygen conditions of the intestinal lumen promote the establishment of an anaerobic microbiota that use fermentation to derive energy from available amino acids and complex polysaccharide (139). Hydrogen sulfide is produced as a byproduct of this fermentation, which is immediately converted to thiosulfate by the epithelial cell layer of the colon (146). During inflammation, neutrophils infiltrate the intestinal lumen and release reactive oxygen species molecules as part of the mechanism for the extracellular killing of bacterial pathogens (146). The association of reactive oxygen species with thiosulfate molecules results in tetrathionate, which can be used by NTS as an electron acceptor during anaerobic respiration. In addition to activating a metabolic response that promotes growth of the pathogen, anaerobic respiration further allows NTS to utilize carbon sources that would otherwise metabolize poorly during aerobic fermentation (147). Altogether, NTS can use these mechanisms to promote their own growth at the expense of the existing host microbiota.

Two features that distinguish enteric or typhoid fever from gastroenteritis are the relative absence of inflammation and an innate immune response, and the replication of typhoidal *Salmonella* in the systemic compartment of the host. These changes in pathogenesis are linked to genomic differences between typhoidal and NTS. Approximately 200 functional genes in NTS have been inactivated or functionally disrupted in *S*. Typhi and *S*. Paratyphi A (148). Many of the mutations in *S*. Typhi affect processes used by NTS to induce intestinal inflammation, including motility and chemotaxis, adherence to and invasion of host cells, and loss of virulence factors associated with intracellular replication (148). The loss of these functions suggest that *S*. Typhi may gain access to the systemic compartment through a mechanism distinct from active invasion of intestinal epithelial cells. Although this mechanism remains elusive, it is hypothesized that microfold (M) cells that sample the intestinal lumen actively take in *S*. Typhi cells and transfer them to macrophages and dendritic cells within the gut-associated lymphoid tissue of the Peyer’s patches, located in the small intestine (16). Typhoidal *Salmonella* are unique/distinct from non-typhoidal serovars in that they are able to persist in this intracellular niche without activating the immune response and infiltration of neutrophils that would otherwise restrict typhoidal infections (149). Within these immune cells, typhoidal *Salmonella* are shuttled to other sites in the body associated with the mononuclear phagocyte system (previously known as the reticuloendothelial system), taking residence in such places as the liver, spleen, mesenteric lymph nodes, bone marrow, as well as the gall bladder (17, 20). Typhoidal *Salmonella* are thought to transfer back into the duodenum *via* the biliary tract, resulting in intermittent shedding of typhoidal cells in the feces (17).

The lack of inflammation associated with typhoidal *Salmonella* infections suggests important differences in the pathogen’s surface antigens or its interactions with host cells. For example, the potential downregulation in serovar Typhi flagellar expression results in decreased inflammation (150). Other gene mutations identified in *S*. Typhi include regulatory elements affecting the O-antigen structure, which may limit exposure of this important PAMP to immune cells (151). Further, the typhoidal *Salmonella* genome also possesses 300 to 400 unique genes that are absent in NTS. Of these additional accessory genes, the Vi capsule plays an important role in reducing the host inflammatory response to the presence of *S*. Typhi cells. Production of the Vi capsule limits complement deposition on the surface of *S*. Typhi cells, masks surface antigens that would normally activate the host immune response, and provides resistance to phagocytic killing (16, 17, 152). Further, the Vi capsule has also been demonstrated to induce production of the cytokine interleukin 10, an important anti-inflammatory molecule (153). Vi-negative mutants of *S*. Typhi were unable to cause enteric fever in human infection trials (154). However, the Vi capsule cannot solely account for differences between typhoidal and NTS infections, as this capsule is not expressed by other typhoidal serovars (i.e., *S*. Paratyphi A) (149). It is likely that serovar-specific combinations of gene acquisition and gene loss are responsible for the ability of typhoidal *Salmonella* strains to evade the host immune response. There are several other reasons for the host-restriction of serovar Typhi strains, as recently discovered by Spanò et al. (155). Further study of these factors will provide insight for understanding how *Salmonella* serovars and strains progress from host-generalists to host-adapted and finally to becoming host restricted.

## Our Current Understanding about *Salmonella* Transmission

Despite their differences in host range, non-typhoidal and typhoidal *Salmonella* serovars maintain a genetic relatedness at the species level (149). Therefore, *Salmonella* pathogens present an opportunity to study the biological factors that are important for transmission (156).

Nearly all NTS serovars associated with human disease demonstrate the ability to colonize multiple host species and induce gastroenteritis. In North America, NTS infections are often associated with the ingestion of contaminated food or water, but can also be transmitted directly from zoonotic sources, such as domestic or food animals, through the fecal–oral route (84). Outbreaks of NTS have been associated with a wide range of food products, including animal-based (meat, poultry, eggs), plant-based (tomatoes, sprouts, melons, lettuce, mangoes, raw almonds) and processed foods (powdered infant formula, dry seasonings, cereals, peanut butter) (84). However, a number of NTS outbreaks in developed countries emphasize the importance of environmental reservoirs as an intermediary step for transmission of this pathogen. Several reported cases exemplify the ability of NTS to persist in the non-host environment. Surface runoff contaminated with animal feces was suspected as the source of two separate cases in 2008 of drinking water contamination affecting communities in the United States (157, 158). Similarly, a study analyzing 288 cases of drinking water-related outbreaks in Canada between the years 1971 and 2001 noted water treatment practices and nearby wildlife as the most frequently reported sources of contamination (159). Several reports of NTS outbreaks associated with fresh produce have traced contamination to irrigation water or animal manure used to fertilize fields (160–162). Of particular interest are two separate outbreaks of NTS infections in the United States in 2002 and 2005, both of which were linked to a rare strain of *S*. Newport in tomatoes (162). In both outbreaks, investigators were able to trace back the unique strain to a Virginian farm, where the strain had been isolated from a contaminated pond used to irrigate the fields (162). The identification of this same rare strain in pond water samples taken years apart indicates the added importance of *Salmonella* persistence in environmental reservoirs. A similar case of non-typhoidal *S*. Typhimurium persistence was observed for a Danish pig farm associated with recurring infections in its herd (163). Samples collected by Baloda and colleagues over a 2-year period revealed the presence of the same *Salmonella* clone in the piggery, the feed provided to the animals, and in the pig manure used to fertilize agricultural soil (163). Farmland soil treated with manure yielded viable *S*. Typhimurium cells for 14 days following spread, providing further evidence for the survival of NTS within the non-host environment. The authors hypothesized that the persistence of *Salmonella* in this setting could result in a cycle of re-infection of the pig herd from environmental reservoirs, potentially explaining the long-term presence of NTS at the farm. While it may be logical to infer a role for biofilm formation in such cases of *Salmonella* persistence, it will be important for future research endeavors to include efforts to characterize the physiological state of *Salmonella* cells *in situ*.

For typhoidal *Salmonella*, chronic persistence within their current host increases the opportunities for subsequent transmission events. Between 5 and 10% of patients recovering from enteric fever experience a relapse in infection with the same typhoidal *Salmonella* strain, resulting in milder symptoms than before and fecal shedding of the pathogen for 3 weeks to 3 months following the initial infection (17). While the mechanism behind this short-term persistence in the host is unclear, it is hypothesized that typhoidal *Salmonella* can remain dormant within immature immune cells in the bone marrow (16). Between 2 and 4% of people living in areas endemic for enteric fever are associated with a chronic carrier state that involves asymptomatic carriage of typhoidal *Salmonella* for more than a year (16). Epidemiological studies hoping to identify factors associated with the chronic carrier state are difficult due to the asymptomatic nature of infections in these hosts (17). However, recent evidence points to persistence of typhoidal *Salmonella* within the gall bladder. It is currently hypothesized that typhoidal *Salmonella* cells first localize to the liver and replicate in the resident macrophage (Kupffer) cells before traveling to the gall bladder *via* the biliary tract (17). Bile, a digestive secretion with detergent and antimicrobial properties, contributes to the sterility of the gall bladder. In a landmark study assessing the incidence of gall bladder disease in patients associated with acute or chronic infections with typhoidal *Salmonella*, the authors detected gallstones in nearly 90% of chronically infected patients (164). Microscopic analysis of this interaction suggested that typhoidal *Salmonella* cells use fimbrial protein structures on their surface to attach to gallstones, while growth in the presence of bile stimulates the production of protective extracellular polysaccharides (59). It is currently hypothesized that short-term human carriers are mainly responsible for the transmission of enteric fever in endemic areas, while long-term chronic carriers are responsible for resurgence of infections in endemic regions despite attempts to control such infections (17). Blaser and Kischner proposed that Typhi carriers would have been necessary in hunter gatherer societies to allow the disease to spread to others even after everyone in the local family group had been infected and had acquired immunity (165). Typhoidal *Salmonella* serovars are transmitted primarily from person to person through food and water contaminated with human feces; as such, infections are more frequent in low-income countries that lack available safe water resources and have poor sanitation standards (166). Cases of typhoidal infections in high-income countries are usually the result of patients traveling to endemic areas, but can also be spread by individuals that are chronically infected with *S*. Typhi (166).

Like typhoidal serovars, strains of iNTS induce a fever-like illness and persist within the systemic compartment of infected individuals (19). As of today, humans are the only identified reservoir for invasive strains of NTS (167, 168). Thus, the speculation is that these strains are transmitted similarly to Typhi, with the human carrier being the main source of new infections. iNTS infections occur predominately in children between 6 and 18 months of age and in adults between 25 and 40 years old (166). For children, the predominant host risk factors are HIV infection, malnutrition, and malaria, while advanced HIV infection is the main risk factor in adults (19). Further, cases of iNTS disease in children and adults are strongly correlated with the rainy season in sub-Saharan Africa, which could be the result of waterborne transmission, malaria, or malnutrition during this season (20, 166).

### Bistable Gene Expression and a New Perspective on Biofilm Formation

Our current understanding of *Salmonella* biofilm formation has been primarily shaped by its characterization in the laboratory. Most early comparisons between biofilm-positive and biofilm-negative isolates were done at a population level. While this yielded valuable information, it was hard to envision how these biofilm phenotypes would manifest in nature. For example, Serra et al. showed that there was massive cell heterogeneity within rdar colonies (40), therefore, ascribing functions to the rdar colony as a whole may not be biologically accurate. Furthermore, this density of cells (i.e., 10^10^–10^11^ cells per colony) would not be sticking together in the environment in one piece, except under very special circumstances, such as a wastewater treatment plant (169), but even in those conditions there are many different species involved so the dynamics would be different. This led us (and others) to the question: “How does the rdar morphotype appear in nature?” To simulate the environment, we attempted to induce *S. enterica* serovar Typhimurium aggregation at low cell densities in liquid cultures (45). Under these conditions, the population of cells in liquid culture differentiated into two forms: multicellular aggregates and planktonic cells. We showed that the aggregates produced curli and cellulose polymers and had many of the properties of a rdar biofilm (45). We know now that the biofilm cells and single cells arise due to bistable production of CsgD (i.e., biofilm cells are CsgD-ON; single cells are CsgD-OFF) (170) (Figure 3B).

In a large-scale RNA-seq experiment, we compared the gene expression profile of *Salmonella* multicellular aggregates and planktonic cells (103). Although they are formed under the same growth conditions, these clonal cell types had differential expression of over 1,856 genes, which represents approximately 35% of all genes in the serovar Typhimurium genome. Previous work had identified genes corresponding to carbon central metabolism and the general stress response being expressed during biofilm formation (54). Transcriptome analysis expanded this by identifying increased expression of genes important for the metabolism of amino acids, lipids, and nucleotides. We demonstrated that biofilm cells were more resistant to desiccation and antibiotics than the planktonic cells. The transcriptome of planktonic cells was vastly different from multicellular aggregates, with significant expression of multiple virulence traits, including the T3SS-1. In the literature, it was thought that expression of the T3SS-1 was exclusively induced by *in vivo* conditions inside the host, which is an important first step in *Salmonella* pathogenesis (133). As such, expression of T3SS-1 in biofilm-inducing conditions was highly unexpected and required rigorous functional validation. We confirmed that the proteins for the secretion apparatus and its effectors were synthesized under these atypical environmental conditions. The increased abundance of T3SS-1 provided a virulence advantage for planktonic cells compared to multicellular aggregates both for invasion of a human intestinal cell line *in vitro* and during competitive infections in mice. Determining how the T3SS-1 is induced in the planktonic cell subpopulation remains an important question for us. The relative absence of SPI-1 expression in multicellular aggregates may also provide an important clue for the molecular link between the persistence and virulence phenotypes. While it is tempting to speculate that there is a direct link between CsgD and SPI-1 expression, as implied by other studies (171), such a relationship has not yet been established. It is plausible that the presence of the extracellular matrix may provide an important feedback signal that ultimately inhibits the expression of virulence factors such as the T3SS-1. Regulation between SPI-1 and biofilm formation may also be indirect in nature. Desai and colleagues recently demonstrated that SsrB, a transcriptional regulator encoded within SPI-2, can switch between promoting expression of the T3SS-2 within the acidic macrophage vacuole and relieving H-NS silencing of *csgD* expression (172). Establishing the genetic link between persistence and virulence is an important direction for the future of *Salmonella* biofilm research.

## Selection Pressures Acting on Biofilm Formation

### Immune Avoidance or Continued Transmission Success?

As described in Section “Correlation between Biofilm Formation and Host Specificity,” biofilm formation is highly conserved in *Salmonella* strains associated with gastroenteritis, but is lost in *Salmonella* strains that are responsible for invasive disease or are adapted to life in a particular host. Romling and colleagues were the first to discuss this correlation in the context of *Salmonella* biology (121). They suggested that loss of biofilm formation in invasive strains/serovars/species was a pathoadaptive trait, presumably to improve the fitness of the pathogen so that it is able to survive better in host tissues (173). Part of the reasoning behind this was the knowledge that *Shigella* and enteroinvasive *E. coli*, two pathogens which breach the intestinal epithelium (174), have lost the rdar morphotype due to multiple insertions and deletions in the curli biosynthesis operons, indicating strong selection pressure against this phenotype (175). In contrast, *E. coli* strains that were commensal inhabitants of the GI tract appeared to retain an ability to express the rdar morphotype (112). We reported the same trend in *E. coli* as in *Salmonella*; among 284 *E. coli* isolates from diverse host species, we observed that host-generalist isolates were 84% rdar-positive, whereas isolates that had the largest genetic differences and were the most likely to be host-adapted were less than 50% rdar-positive (176). The 115 human isolates that we screened were only 36% rdar-positive versus 169 isolates from different animal hosts that ranged from 59 to 93% rdar-positive. We reasoned that human commensal *E. coli* were more host-adapted and would have less reliance on transmission *via* the environment as compared to transient strains that cause extra-intestinal infections and are only in the host for a short time. However, reduced rdar prevalence in our study was also correlated with an increase in the presence of virulence genes, meaning that the dynamics of *E. coli* colonization are complex (176, 177). The possibility of a pathoadaptive trait conserved between species indicates that similar selection pressures are acting on *Salmonella* and *E. coli*. Römling et al. focused on the interactions with the intestinal epithelium, arguing that when a pathogen crosses this barrier the dominant selection pressure comes from the host immune system (121). While the immune system undoubtedly plays an important role, there are also strong pressures on pathogens to maintain their transmission cycles (156). Therefore, loss or impairment of the rdar morphotype could also reflect a change in strain transmission patterns (125). At present, we do not know which aspect of the *Salmonella* lifestyle has the greatest evolutionary influence on the relationship between host adaptation and ability to produce rdar biofilms.

## Conclusion and Predictions

Bistable CsgD expression and analysis of the aggregate and planktonic cell subpopulations has shifted our perception of *Salmonella* biofilm formation and the process of pathogen transmission. What once was considered a population-level phenotype is now understood as a regulatory phenomenon mediated at the single cell level (170). What is the purpose of this phenotype switching in the life cycle of *Salmonella*? Is there an advantage for *Salmonella* to express virulence factors in a non-host setting? Compared to the host niche, where the conditions of host–pathogen interactions are relatively defined, life in non-host environments and the process of transmission are unpredictable. In bacteria, bistable genetic networks are often associated with the formation of two distinguishable phenotypes within a clonal population (178), which is thought to allow genotypes to persist in fluctuating environments (179). Based on this theory and our characterization of the planktonic and aggregated cell subpopulations, we hypothesize that “phenotype switching” improves the overall chances for *Salmonella* transmission. In a scenario where *Salmonella* immediately encounters its next host, planktonic cells would be able to instigate a new infection. In contrast, if a host were not encountered, *Salmonella* biofilm cells would be prepared to survive in non-host environments for a long time until an opportunity for infection arises. This unpredictable step in the *Salmonella* life cycle places equal selection pressure on virulence and persistence phenotypes.

Since non-host environments have unpredictable conditions, it would be a poor evolutionary choice for *Salmonella* cells to adapt once they arrive there. Rather, *Salmonella* likely requires anticipatory genetic regulation where cells pre-emptively express the phenotype that is necessary for the next step in their life cycle (180). If bistable CsgD expression is necessary for cells to prepare for transmission, conditions within the GI tract may provide important cues to induce this differentiation. In the GI niche, *Salmonella* cells are exposed to host temperatures, low pH, high osmolarity, bile acids, antimicrobial peptides, iron limitation, and in some cases, nutrient limitation (141, 181). Some of these conditions (i.e., temperature and osmolarity) may favor the expression of virulence factors such as the T3SS-1 (141), while repressing *Salmonella* biofilm formation (33, 49). Conversely, exposure to stresses such as bile, antimicrobial peptides, iron limitation, or poor nutrient availability would promote *csgD* expression and the biofilm phenotype. Regulation of *csgD* expression and synthesis is incredibly complex, and few studies have attempted to understand the hierarchy of this regulation (33, 49, 77, 182). For example, iron limitation has been shown to over-ride the normal temperature shut-off of biofilm formation at 37°C (33). It is possible that microenvironments within the intestinal niche provide strong activating signals required to generate the CsgD-ON state. In the lumen of the small intestine, high bile concentrations have been shown to increase the intracellular concentration of cyclic-di-GMP in the enteropathogen *V. cholerae* [reviewed in Ref. (181)] and to influence biofilm formation (183). For *Salmonella*, high concentrations of c-di-GMP activate *csgD* expression and promote the biofilm phenotype (51). Expression of the T3SS-1 is known to be location-dependent; *Salmonella* cells positioned at the surface of the intestinal epithelial layer were 100% positive for T3SS-1 expression, while the majority of cells in the lumen were negative for this virulence trait (184). We predict that biofilm expression may also be induced by signals produced toward the end of host–pathogen interactions. These signals may include intestinal inflammation and the associated rapid replication of *Salmonella* within the lumen (139, 146, 147, 185). The reactive oxygen species produced by neutrophils undoubtedly provides an important cue for activation of the bacterial cell stress response. Metabolic cues, such as the pathways involved with anaerobic respiration during this stage [i.e., ethanolamine and 1,2-propanediol (186)] or the nutrient limitation caused by rapid *Salmonella* replication following inflammation, may also add a temporal element to the anticipatory regulation of *Salmonella* biofilm formation. Consistent with this, our transcriptomic analysis revealed that genes for the ethanolamine and 1,2-propanediol metabolic pathways have increased expression within the CsgD-ON multicellular aggregates (103).

Our ability to elucidate the *in situ* regulation of *Salmonella* biofilm formation is limited by the reality that *Salmonella* biofilms have yet to be observed in nature. However, the enteric bacteria *V. cholerae* has been shown to exist both as planktonic cells and in multicellular aggregates in human stool samples (187, 188). We previously investigated *csgD* expression and the biofilm phenotype in *Salmonella* during murine infection by using luciferase reporters fused to biofilm-related gene promoters. *csgD* expression was activated within the mouse intestine during the course of infection, but the expression of curli biosynthesis genes (*csgBAC*) was only observed within fecal pellets that had passed out of the infected mice (45). From this experiment, we concluded that synthesis of the extracellular matrix only occurs after passage from the host. However, we have since learned that there is a limitation in the ability to detect bacterial luciferase during the course of infection (189). Heavy bacterial loads were required for consistent luciferase detection, despite expression from a strong constitutive promoter. This means that if activation of extracellular matrix production was limited to only a small subpopulation of cells as part of CsgD bistability, signaling from the *csgBAC* promoter would not be detectable using bioluminescence as a marker. Work in our laboratory is currently underway to determine if curli fimbriae and multicellular aggregates can be observed during murine infections by using single cell detection techniques (i.e., tissue sectioning and confocal microscopy).

The lifecycle of *Salmonella* involves exposure to both the host and natural (extracorporeal) environments (190). Evidence from our own and other research groups suggests that the rdar biofilm morphotype controlled by CsgD is crucial to the transmission success of *Salmonella*, allowing cells to survive the natural environment that is encountered between host infections (45, 77, 83, 103). Similarly, it may be possible that *Salmonella* produces a cellulose-based biofilm during host infection to mitigate stresses that arise from host immune responses or the harsh intracellular setting of a macrophage (111, 114). While the c-di-GMP signaling network is important for the production of either type of biofilm, most of the enzymes responsible for synthesizing c-di-GMP are exclusively expressed and activated in either a CsgD-dependent or -independent manner (44, 110, 191). Further research is necessary to not only identify the host sites and signals that activate the production of cellulose biofilm during infection, but to determine the reasons why cellulose biofilm production is important in host–pathogen interactions. It is possible that the c-di-GMP-specific pathway for cellulose biofilm expression has evolved to reduce the severity of its virulence and increase the possibility for *Salmonella* to successfully transmit to a future host.

**Figure 4:**
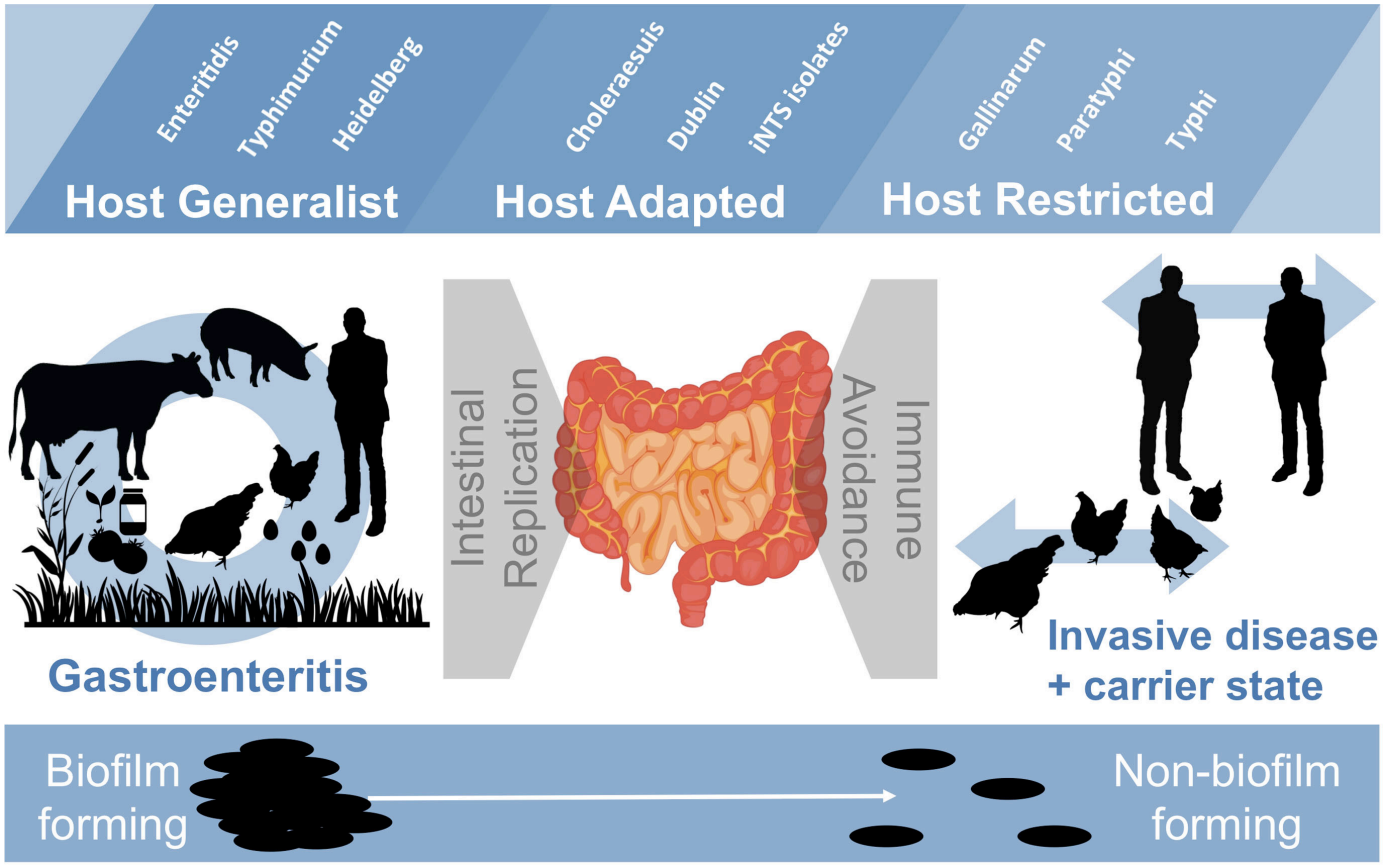
Host interactions, lifecycles, and biofilm-forming ability of *Salmonella* strains. Host-generalist *Salmonella* strains have a varied lifecycle, in which several host species and environments are encountered, and zoonotic transfer to humans may occur. Transfer may also occur through ingestion of contaminated vegetables (i.e., tomatoes, sprouts) or processed foods. Infections are localized to the intestine and the selection pressures are on intestinal replication and transmission. In contrast, host-adapted and host-restricted *Salmonella* strains have an evolutionary narrowed lifecycle, in which transmission is primarily between individual hosts. Selection pressures are on immune avoidance with the objective of long-term persistence within the host. The loss of biofilm formation in host-adapted and host-restricted strains is thought to reflect a shift in selection pressures caused by a change in lifecycle.

In this review, we have touched upon new aspects of *Salmonella* biology that may have important implications for understanding transmission patterns. NTS strains that briefly colonize the host and cause gastroenteritis must contend with survival in both host and non-host environments. On the one hand, they are adapted to colonize the intestines of many different host species and to replicate to high numbers within the inflamed intestinal environment. Perhaps through biofilm formation and the bistable expression of CsgD, the formation of specialized subpopulations of cells (i.e., multicellular aggregates and planktonic cells) represents an evolutionary trade-off for mitigating the unpredictable nature of transmission *via* the fecal–oral route. In contrast, invasive strains of non-typhoidal and typhoidal *Salmonella* have evolved to avoid the immune system in order to persist chronically within the systemic niche of the host. This feature of host adaptation relieves the selection pressure placed on *Salmonella* to persist within an environmental reservoir and correlates with loss of the biofilm phenotype. It could also reflect that loss of biofilm phenotypes relieves selection pressure from the host immune system, which would contribute to their ability to persist *in vivo* (Figure 4).

Recent advances in DNA sequencing technology have expanded the availability of pathogen genomic sequences, which has led to unprecedented characterization of both classical and emerging *Salmonella* strains. iNTS strains are an example of how genome sequencing can be complemented by our understanding of *Salmonella* pathogenesis to develop a genetic signature for host adaptation. Similarly, future research efforts are needed to isolate the core genetic processes that govern biofilm formation. We predict that a biofilm gene signature or gene expression profile could be used to predict the transmission properties of *Salmonella* strains associated with future outbreaks. Understanding common reservoirs and transmission routes is crucial for developing effective public health efforts to reduce the worldwide disease burden of *Salmonella* pathogens.

## Author Contributions

KM and MP prepared the figures, KM and AW wrote the paper, and MP and WK edited the paper. All authors read and approved the final manuscript.

## Conflict of Interest Statement

The authors declare that the research was conducted in the absence of any commercial or financial relationships that could be construed as a potential conflict of interest.
